# Supplementary material for: Development and characterization of two equine formulations towards SARS-CoV-2 proteins for the potential treatment of COVID-19
Source: Sci Rep. 2021 May 10;11:9825. doi: 10.1038/s41598-021-89242-z (PMC8110969; doi:10.1038/s41598-021-89242-z)
Supplement: Supplementary file 1 — Supplementary Fig. S1. [file 41598_2021_89242_MOESM1_ESM.docx]

**SUPPLEMENTARY INFORMATION**

**Development and characterization of two equine formulations towards SARS-CoV-2 proteins for the potential treatment of COVID-19**

Guillermo León^1^, María Herrera^1^, Mariángela Vargas^1^*, Mauricio Arguedas^1^, Andrés Sánchez^1^, Álvaro Segura^1^, Aarón Gómez^1^, Gabriela Solano^1^, Eugenia Corrales-Aguilar^2^, Kenneth Risner^3^, Aarthi Narayanan^3^, Charles Bailey^3^, Mauren Villalta^1^, Andrés Hernández^1^, Adriana Sánchez^1^, Daniel Cordero^1^, Daniela Solano^1^, Gina Durán^1^, Eduardo Segura^1^, Maykel Cerdas^1^, Deibid Umaña^1^, Edwin Moscoso^1^, Ricardo Estrada^1^, Jairo Gutiérrez^1^, Marcos Méndez^1^, Ana Cecilia Castillo^1^, Laura Sánchez^1^, Ronald Sánchez^1^, José María Gutiérrez^1^, Cecilia Díaz^1^, Alberto Alape^1,4^

^1^Instituto Clodomiro Picado, Facultad de Microbiología, Universidad de Costa Rica, San José, Costa Rica. ^2^Virology-CIET (Research Center for Tropical Diseases), Microbiology, University of Costa Rica, San José, Costa Rica. ^3^National Center for Biodefense and Infectious Diseases, George Mason University, Virginia, United States of America. ^4^ Departamento de Bioquímica, Escuela de Medicina, Universidad de Costa Rica, San José, Costa Rica.

*Email: [mariangela.vargasarroyo@ucr.ac.cr](mailto:mariangela.vargasarroyo@ucr.ac.cr)


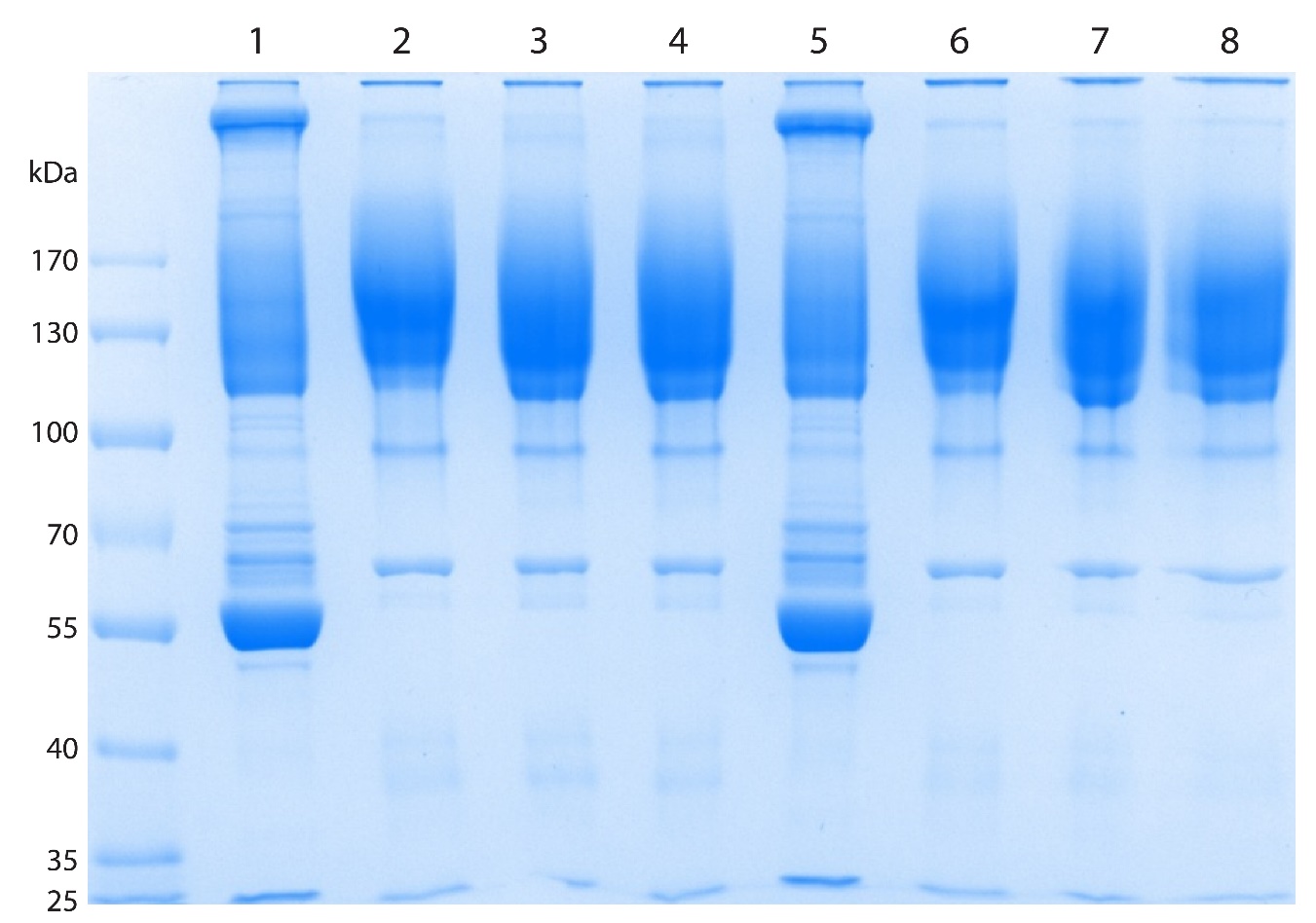


**Figure S1**. SDS-PAGE analysis of pools of plasma and final anti-S1 and anti-Mix formulations. Lane 1: Hyperimmune pool of plasma anti-S1; Lanes 2-4: Anti-S1 formulation; Lane53: Hyperimmune pool of plasma anti-Mix; Lanes 6-8: Anti-Mix formulation. Samples (20 µg) were loaded in a 7.5% polyacrylamide gel in the presence of SDS and run under non-reducing conditions. The gel was stained with Coomassie Blue.
